# Inequities in Availability of Evidence-Based Birth Supports to Improve Perinatal Health for Socially Vulnerable Rural Residents

**DOI:** 10.3390/children9071077

**Published:** 2022-07-19

**Authors:** Bridget Basile Ibrahim, Julia D. Interrante, Alyssa H. Fritz, Mariana S. Tuttle, Katy Backes Kozhimannil

**Affiliations:** 1 University of Minnesota Rural Health Research Center, Division of Health Policy and Management, School of Public Health, University of Minnesota, Minneapolis, MN 55414, USA; inter014@umn.edu (J.D.I.); ahfritz@umn.edu (A.H.F.); tuttl090@umn.edu (M.S.T.); kbk@umn.edu (K.B.K.); 2 School of Nursing, Yale University, Orange, CT 06477, USA

**Keywords:** rural, maternal health inequities, evidence-based care, health equity, social vulnerability, maternal mortality, maternal morbidity, infant mortality, midwifery, breastfeeding, birth, obstetric

## Abstract

Rural residents in the United States (US) have disproportionately high rates of maternal and infant mortality. Rural residents who are Black, Indigenous, and People of Color (BIPOC) face multiple social risk factors and have some of the worst maternal and infant health outcomes in the U.S. The purpose of this study was to determine the rural availability of evidence-based supports and services that promote maternal and infant health. We developed and conducted a national survey of a sample of rural hospitals. We determined for each responding hospital the county-level scores on the 2018 CDC Social Vulnerability Index (SVI). The sample’s (*n* = 93) median SVI score [IQR] was 0.55 [0.25–0.88]; for majority-BIPOC counties (*n* = 29) the median SVI score was 0.93 [0.88–0.98] compared with 0.38 [0.19–0.64] for majority-White counties (*n* = 64). Among counties where responding hospitals were located, 86.2% located in majority-BIPOC counties ranked in the most socially vulnerable quartile of counties nationally (SVI ≥ 0.75), compared with 14.1% of majority-White counties. In analyses adjusted for geography and hospital size, certified lactation support (*a*OR 0.36, 95% CI 0.13–0.97), midwifery care (*a*OR 0.35, 95% CI 0.12–0.99), doula support (*a*OR 0.30, 95% CI 0.11–0.84), postpartum support groups (*a*OR 0.25, 95% CI 0.09–0.68), and childbirth education classes (*a*OR 0.08, 95% CI 0.01–0.69) were significantly less available in the most vulnerable counties compared with less vulnerable counties. Residents in the most socially vulnerable rural counties, many of whom are BIPOC and thus at higher risk for poor birth outcomes, are significantly less likely to have access to evidence-based supports for maternal and infant health.

## 1. Introduction

Many clinical services, care resources, and community supports [1] have strong evidence showing that they improve maternal and infant health outcomes. These evidence-based supports for maternal and infant health (hereafter evidence-based supports) improve health outcomes in a variety of ways. Having access to the full spectrum of perinatal care (prenatal and postpartum care) [2,3], perinatal mental health services [4,5,6,7], and formal lactation support from international board-certified lactation consultants (IBCLC) [8,9,10,11]) close to home, or even in the home with nurse home visiting [12,13,14,15], helps ensure that pregnant women and people get the screenings, clinical guidance, and monitoring they need to have the healthiest pregnancy and best outcomes possible. Models of care that are person- or family-centered, such as perinatal care with certified nurse-midwives [16,17], prenatal care offered in a group setting (often called group prenatal care) [18,19], and dedicated birth support from doulas (trained non-clinical birth support personnel) [20,21] have been associated with improved maternal and infant health outcomes, particularly for families who are at higher risk for poor outcomes [17,22,23,24,25,26]. Community supports in the form of postpartum [27] and breastfeeding peer support groups [27,28,29], childbirth education classes [30], and nutrition support programs for vulnerable families (Special Supplemental Nutrition Program for Women, Infants, & Children-WIC) [31,32] also have strong evidence in the literature for improving maternal and infant health outcomes.

Social vulnerability encompasses many social and structural determinants of health and health inequities such as household income, employment, education, health insurance, majority (English) language ability, housing, and transportation [33,34,35,36,37,38]. Race and ethnicity, a variable considered in the calculation of social vulnerability [38,39] can be considered as a proxy for exposure to racism [40,41], which impacts health when experienced both interpersonally and due to the upstream effects of social and political structures within society (i.e., institutional and structural racism) [36,42,43]. High social vulnerability has been linked to inequities in maternal and infant health outcomes, including delayed diagnosis of congenital heart anomalies and a resulting higher rate of infant mortality [44], increased preterm birth rates [45], higher teen birth rates [46], and COVID-19-related maternal and infant complications [47]. Specific social determinants of health that are components of social vulnerability have also been linked to adverse maternal and infant outcomes. Previous research has noted associations between: socioeconomic status with maternal health outcomes [35]; insurance status, adequate housing, and transportation with prenatal and postpartum care utilization [15,48]; and educational attainment and neighborhood quality with preterm birth [49].

Rural residents account for 15% of annual US births [50] and are disproportionately affected by poor maternal and infant outcomes [51,52,53,54,55]. Individuals who have intersecting identities that are both rural and Black, Indigenous, or People of Color (BIPOC) have some of the worst birth outcomes in the country [56,57]. In order to improve these outcomes, it is essential to first understand the landscape of available perinatal health services in rural areas. While prior research has noted that access to midwifery services [58,59] and lactation support [60,61,62] in rural US communities is limited, very little is known about the local availability of other evidence-based supports for maternal and infant health for rural families, especially contextualized by community social vulnerability. The purpose of this analysis is to describe the availability of these evidence-based supports (local access to care, family-centered models of care, peer and community supports for families, and health-focused programming) in rural communities that have hospital-based inpatient labor and birth services, with a focus on social vulnerability at the intersection of race/ethnicity and geography.

## 2. Materials and Methods

Study methods have been described in detail elsewhere [63]. In brief, we identified rural hospitals that were operating an inpatient labor and birth in 2018 based on an enhanced two-stage method [64]. Along with a random sample of hospitals in rural counties where the majority of the population is White, non-Hispanic (*n* = 200), we sampled all hospitals located in rural counties where the majority (≥50%) of residents are BIPOC (*n* = 110) due to documented disparities in maternal and infant health outcomes [50,51,52,53,54,55,56,57,65]. Our team confirmed via hospital website and/or telephone calls whether hospitals still offered inpatient care for labor and birth. Those that did not were removed, bringing the final sample to 285 rural hospitals located in all four US Census Regions [66].

A 47-question web-based survey was developed with expertise from rural clinicians and administrators and piloted with administrators at six rural hospitals [63]. Questions were edited for clarity after pilot review. We administered the survey from March to August 2021 via the Qualtrics (Provo, UT, USA) platform. Twelve questions asked about the availability of evidence-based supports in each hospital’s rural community, including: traditional model/individual prenatal care, nurse home visiting services in the prenatal and postpartum periods, perinatal mental health services, lactation support from IBCLC, maternity care with certified nurse-midwives, group prenatal care, birth support from doulas, postpartum peer support groups, breastfeeding support groups, childbirth education classes, and the Special Supplemental Nutrition Program for Women, Infants, & Children (WIC).

We emailed a letter describing our study to hospital chief executive officers (CEO) and chief nursing officers (CNO) and asked the CEO/CNO to forward the Qualtrics survey link to the nurse manager of their labor and birth unit. Follow-up efforts consisted of contacting hospitals by telephone, sending reminder emails to hospital leadership points-of-contact, and mailing postcards with a scannable Qualtrics survey QR code directly to the nurse managers of the labor and birth units.

### 2.1. Exposure: Social Vulnerability Index 2018

The Social Vulnerability Index (SVI), compiled and calculated by the Centers for Disease Control and Prevention (CDC), is a relative measurement of vulnerability, resources, and disadvantage [37,38] that encompasses four themes: (1) socioeconomic status, (2) household composition and disability, (3) minority status and language, and (4) housing and transportation [38]. SVI scores are reported at the county and census tract level as an overall score for vulnerability and a score for each of the four themes. While the SVI was initially intended to identify the most vulnerable areas during public health and natural disaster emergencies, it has also been used for public health/epidemiology research as an objective measure of neighborhood and county-level social vulnerability and disadvantage [44,46,67,68,69,70].

The SVI is a relative vulnerability score using 15 factors from US Census data. We used county-level SVI linked by county Federal Information Processing System (FIPS) codes as the exposure in this analysis [71]. Scores range from 0 to 1; scores closer to 1 indicate a county’s greater social vulnerability relative to other counties across the United States. The scores for each of the four themes use the census data factors as follows: (1) socioeconomic status: percentage of residents living below the poverty line, those who are unemployed, median household income, and high school diploma attainment for residents >25 years old, (2) household composition and disability: percentage of residents aged <18 years and >64 years, civilians with a disability, and single-parent households, (3) racial and ethnic minority and language: percentage of residents who identify as any race/ethnicity other than White, non-Hispanic, and those who speak English “less than well”, and (4) housing type and transportation: percentage of multi-unit structures, mobile homes, crowded dwellings, lack of household vehicle access, and institutionalized group quarters [37,38,72]. The method for SVI calculation and the complete description of data sources is publicly available from the CDC [37,38,72].

### 2.2. Outcome: Community Availability of Evidence-Based Supports for Maternal and Infant Health

Availability of twelve evidence-based supports was indicated by respondents on the survey. The specific evidence-based supports queried were (1) access to care: traditional model/individual prenatal care, nurse home visiting services in the prenatal and postpartum periods, perinatal mental health services, and lactation support from IBCLCs; (2) family-centered models of perinatal care: maternity care with certified nurse-midwives, group prenatal care, and birth support from doulas; (3) peer and community supports for families: postpartum peer support groups and breastfeeding support groups; and (4) health-focused programming: childbirth education classes and WIC.

Survey respondents indicated whether or not each evidence-based support was available in their respective hospitals’ community by selecting from the following options: “available in the community and affiliated with my hospital”, “available in the community and not affiliated with my hospital”, “not available in the community”, or “I don’t know”. The evidence-based supports described in our results as available in the community are composed of a combination of responding hospitals that indicated “available in the community and affiliated with my hospital” and “available in the community and not affiliated with my hospital”.

### 2.3. Statistical Analysis

Descriptive statistics, including median SVI scores and interquartile ranges (IQR) were calculated. Overall SVI score was dichotomized into two groups: the counties that fell into the most vulnerable quartile of counties on the national SVI scale (SVI score ≥ 0.75) and the remaining counties with less vulnerability (SVI score < 0.75), similar to other work using the SVI [44]. Logistic regression was used to determine the likelihood of availability of each of the evidence-based supports associated with county-level social vulnerability, comparing counties in the most vulnerable SVI quartile to the less vulnerable counties. Unadjusted and adjusted odds ratios and 95% confidence intervals are presented. All adjusted analyses controlled for U.S. Census region [66] and urban adjacency (from the Area Health Resources Files [73]), as well as hospital size (average daily census in 2018 from the American Hospital Association Annual Survey [74]). In order to account for regional variability in licensure, integration, and Medicaid reimbursement and expansion, we controlled for census region. To account for the fact that hospitals in rural areas that are adjacent to urban areas may offer more limited services and supports because they are available in nearby urban areas, we controlled for urban adjacency. Hospital size was controlled for to account for the variability in demand for services as well as capacity to offer them. Statistical analyses were completed using SAS software, Version 9.4 (Cary, NC, USA). This study was deemed exempt by the Institutional Review Board of The University of Minnesota (protocol code 00010917, 6 October 2020).

## 3. Results

### 3.1. Study Sample

Ninety-three hospitals (93/285) completed the survey for a response rate of 32.6% (Table 1). Responding hospitals were located in all four U.S. Census regions [66] with 5.4% located in the Northeast, 31.2% in the Midwest, 27.9% in the South, and 35.5% in the West. Approximately 1 in 3 (31.2%) responding rural hospitals were located in majority-BIPOC counties. Critical Access Hospitals comprised 35.5% of responding hospitals. Responding hospitals had a median of 274 births in 2019 (IQR 120–446) and a median average daily census of 22 (IQR 10–53). The sample is described in full detail elsewhere [63,75].

### 3.2. Social Vulnerability Index

Responding hospitals had a median overall SVI score of 0.55 (IQR 0.25–0.88), a socioeconomic status (theme 1) median score of 0.53 (IQR 0.23–0.92), a household composition (theme 2) score of 0.54 (IQR 0.33–0.82), a BIPOC status and language (theme 3) score of 0.59 (IQR 0.25–0.89), and a transportation and housing (theme 4) score of 0.62 (IQR 0.40–0.84) (Table 2). When the sample was disaggregated by majority county population (majority-BIPOC versus majority-White, non-Hispanic), SVI scores indicated higher social vulnerability for BIPOC counties, with a median score approximately twice that of the majority-White, non-Hispanic counties. Majority-BIPOC counties had a total SVI score of 0.93 (IQR 0.88–0.98), a socioeconomic status (theme 1) median score of 0.89 (IQR 0.75–0.96), a household composition (theme 2) score of 0.82 (IQR 0.54–0.94), a minority status and language (theme 3) score of 0.94 (IQR 0.91–0.96), and a transportation and housing (theme 4) score of 0.84 (IQR 0.61–0.91). In comparison, majority-White, non-Hispanic counties had an overall median SVI score of 0.38 (IQR 0.19–0.64), a socioeconomic status (theme 1) median score of 0.44 (IQR 0.18–0.63), a household composition (theme 2) score of 0.43 (IQR 0.21–0.67), a minority status and language (theme 3) score of 0.38 (IQR 0.19–0.63), and a transportation and housing (theme 4) score of 0.47 (IQR 0.30–0.71).

When the counties were dichotomized into those falling within the highest quartile for vulnerability on the national Social Vulnerability Index, (most vulnerable quartile = SVI score ≥ 0.75), counties with a majority of residents who were BIPOC fell into the most vulnerable quartile on overall SVI and all sub-themes at significantly higher rates than the majority-White counties (Table 3). A total of 34 responding rural hospitals were located in counties that were in the most socially vulnerable quartile, 25 of which were majority-BIPOC counties. For overall SVI score, 86.2% of majority-BIPOC counties were in the most vulnerable quartile compared with 14.1% of majority-White counties. For socioeconomic status (theme 1) 75.9% of majority-BIPOC vs. 14.1% of majority-White counties were in the most vulnerable quartile. For household composition (theme 2), 55.1% of majority-BIPOC counties compared with 15.6% of majority-White counties were most vulnerable. The minority status and language theme (theme 3) measures racial/ethnic composition and is therefore almost entirely overlapping with the comparison of majority-White and majority-BIPOC rural counties; as such, 93.1% of BIPOC counties were most vulnerable, compared with 9.4% of majority-White counties. Finally, on the transportation and housing theme (theme 4), 65.5% of majority-BIPOC counties compared with 21.9% of majority-White counties were in the most vulnerable quartile.

### 3.3. Availability of Evidence-Based Supports for Maternal and Infant Health

Responding hospitals were located in rural communities that lacked many of the investigated evidence-based supports (Table 4). At least half of the communities did not have the following evidence-based supports available: nurse home visiting in the prenatal and postpartum periods, midwifery care, group prenatal care, doula care, and postpartum peer support groups. Availability of evidence-based supports also varied significantly between the most and less socially vulnerable counties. Nurse home visiting in the prenatal period was significantly less available in the most vulnerable communities (24.2% vs. 47.4%; *p* = 0.03), as were lactation support from IBCLCs (48.5% vs. 70.2%; *p* = 0.04), doula care (33.3% vs. 59.7%; *p* = 0.02), postpartum support groups (27.3% vs. 61.4%; *p* < 0.01), and childbirth education classes (75.8% vs. 98.3%; *p* < 0.01). While not significantly different, many additional evidence-based supports were also less available in the most vulnerable counties, including traditional model/individual prenatal care, nurse home visiting in the postpartum period, perinatal mental health services, care from nurse-midwives, and breastfeeding support groups. The most vulnerable counties had greater availability of group prenatal care and WIC programming, but these differences were also not statistically significant.

In logistic regression models adjusted for geography (census region and urban adjacency) and hospital size, the likelihood of availability of many evidence-based supports was significantly lower in the most vulnerable counties compared with the less vulnerable counties (Table 5). Evidence-based supports that were significantly less likely to be available in the most vulnerable counties included: lactation support from IBCLCs (*a*OR 0.36, 95% CI 0.13–0.97), midwifery care from certified nurse-midwives (CNM; *a*OR 0.35, 95% CI 0.12–0.99), doula care (*a*OR 0.30, 95% CI 0.11–0.84), postpartum support groups (*a*OR 0.25, 95% CI 0.09–0.68), and childbirth education classes (*a*OR 0.08, 95% CI 0.01–0.69). WIC availability was nearly ubiquitous and was therefore not analyzed in logistic regression models.

## 4. Discussion

This study highlighted inequities in the availability of evidence-based supports for maternal and infant health for many of the nation’s most vulnerable residents, especially those with intersecting identities of rural resident and BIPOC race/ethnicity. The counties where responding hospitals were located differed in respect to their social vulnerability, with majority-BIPOC rural counties being more socially vulnerable across all dimensions of the SVI when compared to majority-White rural counties. The availability of evidence-based supports, programs and interventions that have been shown to improve maternal and infant health outcomes [2,3,4,5,6,7,8,9,10,11,12,13,14,15,16,17,18,19,20,21,27,28,29,30,31,32] also differed, with more vulnerable counties having significantly less access to these resources. This inequitable access is noteworthy given known health inequities for rural BIPOC birthing people and their infants [56,76].

Structural racism has been associated with poor health in both urban and non-urban areas [57,65] as well as with poorer maternal and infant birth outcomes for Black and Indigenous people [57,77,78,79,80,81]. For example, Black infants in areas with high levels of structural racism have higher odds of preterm birth and infant mortality [57,80,82]. Many of the variables used to calculate the SVI have been identified as indicators of structural racism [43,70,83,84,85]. This analysis showed that majority-BIPOC rural counties had significantly higher rates of social vulnerability across all dimensions, further indicating that structural racism exists in many types of community, including rural spaces. Further, the decreased availability of evidence-based supports in the most vulnerable counties, which tend to be majority-BIPOC, and the inequities in birth outcomes experienced by rural BIPOC families may be evidence of structural racism.

These findings point toward a number of policy implications that may increase access to evidence-based supports to improve rural maternal and infant health. This analysis showed that lactation support from IBCLCs was significantly less likely to be available to birthing people in the most vulnerable rural counties. Breastfeeding rates are lower among Black and Indigenous women and birthing people in the US [86], who were more likely to live in the most vulnerable counties in our study. This study highlighted that WIC, which often provides peer lactation support but not as frequently IBCLC breastfeeding support [87], was available in almost all responding hospitals’ rural counties. If appropriations for WIC were increased, this could potentially increase the capacity of local WIC agencies to offer IBCLC lactation support and breastfeeding support groups in rural BIPOC communities, potentially increasing breastfeeding rates for those most at risk due to social vulnerability [10,11].

Medicaid is an important source of financing for care during pregnancy, childbirth, and postpartum in rural communities [88]. Medicaid policies regarding coverage may impact access to evidence-based supports for Medicaid participants, who by virtue of qualifying for Medicaid would be considered socioeconomically vulnerable. Medicaid coverage, which varies by state, may impact access to many of the resources discussed including midwifery care, doula coverage, group prenatal care, childbirth education classes, visiting nurse services, and breastfeeding support from IBCLC. Additional efforts to increase enrollment of pregnant people who qualify for WIC and Medicaid as early as possible in their pregnancy may further increase early access to evidence-based supports for those who would benefit from them most.

Workforce retention and recruitment programs are an additional policy lever to improving availability of evidence-based supports in rural communities. Workforce policies and programs that could improve access to evidence-based supports and services include: expanding insurance reimbursement for and systemic integration of midwifery services [89], increasing training opportunities in perinatal mental health screening and treatment for a variety of mental health clinicians who are already living and practicing in rural areas (primary care providers, social workers, clinical psychologists, etc.), expanding student loan forgiveness programs for health professionals practicing in rural communities [90], subsidizing training programs for doulas of color, and expanding insurance reimbursement for doula care [91].

## 5. Limitations

This study has a number of limitations. The SVI is the main exposure variable and includes race/ethnicity as a variable in the calculation of overall SVI score and for calculating theme 3; therefore, the overall SVI and theme 3 scores are skewed toward higher scores for majority-BIPOC counties. However, given that the majority-BIPOC counties were significantly more vulnerable in all subthemes, not solely the one that includes race and language variables as indicators, the SVI remains a relevant exposure variable to evaluate relative social vulnerability across the US. Because it was not possible to exclude the minority status (theme 3) portion from the overall SVI score as our outcome, we conducted sensitivity analyses controlling for theme 3 scores, and results were substantively unchanged. This analysis only included rural communities that have an operating hospital with a labor and birth unit and, therefore, did not examine availability of evidence-based supports in rural communities that do not have a hospital with a labor and birth unit, which includes more than half of all rural counties [92]. Given the characteristics of rural counties without hospital-based labor and birth care [50,92,93,94], it is likely that these communities have even less availability of evidence-based supports for pregnancy and childbirth.

Survey responses may be biased toward greater reporting of supports available in the community, as administrators who completed the survey may have felt more confident in their hospital and community resources than those who did not participate. This would likely over-represent support access. Alternatively, because the survey respondents were hospital-based, respondents may not be aware of all supports available in non-hospital settings in the community, which could undercount support. Future research could explore barriers to access to supports available within rural communities and broaden assessment of evidence-based sexual and reproductive supports beyond the pregnancy and postpartum period.

The ongoing COVID-19 pandemic impacted hospital administrator availability to participate in research and may also have influenced responses. Responding and non-responding hospitals were not significantly dissimilar, other than responding hospitals being more likely to be located in the West and less likely to be located in the South [63]. A strength of this study is that it is among the first to collect data about availability of evidence-based maternal and infant health resources from rural communities themselves, and it presents new information on social vulnerability and social determinants of maternal and infant health in rural US counties.

## 6. Conclusions

Rural communities have significant diversity in their social vulnerability, with majority-BIPOC counties having greater vulnerability across all dimensions. The most socially vulnerable rural communities (especially those with a majority-BIPOC population) are also the least likely to have access to community resources to promote maternal and infant health.

## Figures and Tables

**Table 1:** Demographics of responding rural hospitals with labor and birth units, *n* = 93.

	*n* (%) or Median [IQR]
Region	
Northeast	5 (5.4)
Midwest	29 (31.2)
South	26 (27.9)
West	33 (35.5)
Rural county type by urban adjacency	
Micropolitan, adjacent	28 (30.1)
Micropolitan, non-adjacent	32 (34.4)
Noncore, adjacent	18 (19.4)
Noncore, non-adjacent	15 (16.1)
Majority (≥50%) of county population is BIPOC	29 (31.2)
Critical Access Hospital	33 (35.5)
Number of births, 2019, median [IQR]	274 [120–446]
Hospital average daily census, 2018, median [IQR]	22 [10–53]

BIPOC = Black, Indigenous, People of Color.

**Table 2:** Social Vulnerability Index scores of counties with responding rural hospitals with inpatient labor and birth services.

Social Vulnerability Index (SVI) Score	Full Sample, *n* = 93 Median [IQR]	Majority-BIPOC County, *n* = 29 Median [IQR]	Majority-White, NH County, *n* = 64 Median [IQR]
Overall SVI score	0.55 [0.25–0.88]	0.93 [0.88–0.98]	0.38 [0.19–0.64]
Theme 1-Socioeconomic status	0.53 [0.23–0.92]	0.89 [0.75–0.96]	0.44 [0.18–0.63]
Theme 2-Household composition	0.54 [0.33–0.82]	0.82 [0.54–0.94]	0.43 [0.21–0.67]
Theme 3-Minority status/language	0.59 [0.25–0.89]	0.94 [0.91–0.96]	0.38 [0.19–0.63]
Theme 4-Transportation/housing	0.62 [0.40–0.84]	0.84 [0.61–0.91]	0.47 [0.30–0.71]

SVI—Social Vulnerability Index; NH—non-Hispanic.

**Table 3:** County-level social vulnerability (SVI) by racial majority of county population, for counties with responding rural hospitals with inpatient labor and birth services.

	Full Sample, *n* = 93 (%)	Majority-BIPOC County *n* = 29 (%)	Majority-White, NH County, *n* = 64 (%)	*p*-Value
Overall SVI score in most vulnerable quartile ^	34 (36.6)	25 (86.2)	9 (14.1)	<0.01
Theme 1-Socioeconomic status	31 (33.3)	22 (75.9)	9 (14.1)	<0.01
Theme 2-Household composition	26 (28.0)	16 (55.1)	10 (15.6)	<0.01
Theme 3-Minority status/language	33 (35.5)	27 (93.1)	6 (9.4)	<0.01
Theme 4-Transportation/housing	33 (35.5)	19 (65.5)	14 (21.9)	<0.01

*p*-values are chi-square for responding hospitals in majority-BIPOC counties vs. majority-White counties. SVI—Social Vulnerability Index; NH—non-Hispanic. ^ Most vulnerable quartile = SVI score ≥ 0.75 on national scale.

**Table 4:** County-level social vulnerability and availability of evidence-based supports in 93 counties with responding rural hospitals.

	Total, *n* = 90 (%)	Most Vulnerable Quartile, ^ *n* = 33 (%)	Less Vulnerable Quartiles, *n* = 57 (%)	*p*-Value
Local access to care				
Individual (traditional model) prenatal care	85 (94.4)	30 (90.9)	55 (96.5)	0.27
Nurse home visiting-prenatal period	35(38.9)	8 (24.2)	27 (47.4)	0.03
Nurse home visiting-postpartum period	43 (47.8)	12 (36.4)	31 (54.4)	0.10
Perinatal mental health services	59 (65.6)	19 (57.6)	40 (70.2)	0.23
Lactation support from IBCLC	56 (62.2)	16 (48.5)	40 (70.2)	0.04
Family-centered models of care				
Midwifery care with CNM	42 (46.7)	13 (39.4)	29 (50.9)	0.29
Group prenatal care	34 (38.2)	13 (39.4)	21 (37.5)	0.86
Doula care	45 (50.0)	11 (33.3)	34 (59.7)	0.02
Peer and community supports for families				
Postpartum support groups	44 (48.9)	9 (27.3)	35 (61.4)	<0.01
Breastfeeding support groups	72 (80.0)	23 (69.7)	49 (86.0)	0.06
Health-focused programming				
Childbirth education classes	81 (90.0)	25 (75.8)	56 (98.3)	<0.01
Nutrition program (WIC)	88 (97.8)	33 (100)	55 (96.5)	0.28

*p*-values are chi-square for responding hospitals in the most socially vulnerable counties vs. the remainder of counties with responding rural hospitals. IBCLC—International Board-Certified Lactation Consultants; CNM—certified nurse-midwife; WIC—Special Supplemental Nutrition Program for Women, Infants, and Children. ^ Most vulnerable quartile = SVI score ≥ 0.75 on national scale.

**Table 5:** Likelihood of availability of evidence-based birth supports and services in most vulnerable rural counties with rural hospitals with inpatient labor and birth services.

Evidence-Based Support/Service	OR	(95% CI)	*a*OR ^	(95% CI)
Local access to care				
Individual (traditional model) prenatal care	0.36	(0.06–2.30)	0.17	(0.01–2.34)
Nurse home visiting-prenatal period	0.36	(0.14–0.92)	0.41	(0.15–1.15)
Nurse home visiting-postpartum period	0.48	(0.20–1.16)	0.48	(0.18–1.24)
Perinatal mental health services	0.58	(0.24–1.41)	0.67	(0.26–1.74)
Lactation support from IBCLC	0.40	(0.17–0.97)	0.36	(0.13–0.97)
Family-centered models of care				
Midwifery care with CNM	0.63	(0.26–1.50)	0.35	(0.12–0.99)
Group prenatal care	1.08	(0.45–2.62)	1.01	(0.38–2.67)
Doula support	0.34	(0.14–0.83)	0.30	(0.11–0.84)
Peer and community supports for families				
Postpartum support groups	0.24	(0.09–0.60)	0.25	(0.09–0.68)
Breastfeeding support groups	0.38	(0.13–1.08)	0.42	(0.14–1.29)
Health-focused programming				
Childbirth education classes	0.06	(0.01–0.47)	0.08	(0.01–0.69)

Reference = less vulnerable counties (overall SVI < 0.75). ^ adjusted for geography (U.S. census region and urban adjacency) and hospital size (average daily census in 2018). IBCLC—International Board-Certified Lactation Consultants; CNM—certified nurse-midwife; OR—odds ratio; *a*OR—adjusted odds ratio; CI—confidence interval.

## Data Availability

The data used to create the sampling frame for this survey include information that is subject to a data use agreement and cannot be made available to others. The survey instrument is available as an online supplemental document (doi:10.1001/jamahealthforum.2022.0204), and analytic code can be provided by the corresponding author on request.
